# Estimated Daily Urine Volume and Solute Excretion from Spot Urine Samples to Guide the Therapy of Hyponatremia in SIADH

**DOI:** 10.3390/jcm8101511

**Published:** 2019-09-20

**Authors:** Guy Decaux, Wim Musch

**Affiliations:** 1 Department of Internal Medicine Erasmus University Hospital, ULB, B-1070 Brussels, Belgium; 2 Department of Internal Medicine, Bracops Hospital, 1070 Anderlecht, Belgium

**Keywords:** hyponatremia, urea, diuresis, urine osmolality

## Abstract

**Background:** In hyponatremia, due to the inappropriate secretion of antidiuretic hormone (SIADH), a high versus low solute intake will affect the urine volume (UV) and, hence, the SNa level. The clinical implication of the fractional solute excretion is presented. **Methods**: In 35 normal controls and 24 patients with SIADH and urine osmolality higher than serum osmolality, we compared exact solute intake obtained from 24 h urine collection, with the estimated value obtained on a urine morning spot sample by the formula: *eGFR (L/min) × Sosm × 1440 × FE.Osm (%) = mmol/24 h.* The exact UV was compared with the estimated value given by the formula: *eGFR × 1440 × S.Creat/U.Creat (for eGFR the MDRD was used).* In 65 patients with chronic SIADH, from which a morning spot urine sample was available, we determined the estimated fluid and solute intake. **Results**: A good correlation was observed between the measured solute output or urine volume and the estimated values obtained from the controls (*r* = 0.86) as well as in SIADH (*r* = 0.91). **Conclusion**: Patients with low solute intake (FE.Osm <1.4%) and low diuresis (V/eCcr <0.8%) should increase their intake by taking oral urea, for example. Patients with high solute intake (FE.Osm >2.5%) and high diuresis (V/eCcr >1.5%) could usually be treated by mild water restriction (<1.5–21/24 h).

## 1. Introduction

Hyponatremia related to the inappropriate secretion of antidiuretic hormone (SIADH) is one of the most frequent types of hyponatremia. Hyponatremia is mainly due to water retention, and water restriction (WR) is the first approach in treating hyponatremia related to SIADH. Around 20% of patients can be controlled by mild WR [1]. Another way to achieve a negative water balance is to increase diuresis by increasing urine solute excretion [2] or by taking anti-V2 blocking medication [1,3].

In normal healthy adults, the solute output averages 700 mmol/day [4]. About half is represented by electrolytes (and NH_4_+) and the other half by urea. A high solute intake will increase diuresis and help to achieve a negative water balance. In normal conditions, we consider that the urine volume (UV) reflects the fluid intake increased, or not, by about 300 mL [5].

To diagnose hyponatremia due to SIADH, we usually measure osmolality and sodium concentration on a urine spot. Urine osmolality is inappropriate if it is not lower than 100 mOsm/kg H2O in a patient with serum hypoosmolality. Patients with urine osmolality between 100 and 300 mOsm/kg are usually easy to treat by simple water restriction, at least if solute intake is normal in the long term [6].

An easy way to determine solute intake in a patient is to measure the total solute excretion in their urine, given by the urine osmolality multiplied by the 24 h urine volume. This value is highly variable between patients as some of them have a high-protein and high-salt diet, whereas others have their caloric intake represented mainly by a high-sugar and high-fat diet but a low solute excretion because of low meat and salt intakes. In the elderly, a low solute intake is particularly frequent.

Patients with chronic hyponatremia are frequently considered as asymptomatic and are left untreated if WR is not effective. Although hyponatremia is associated with increased falls, attention deficit, gait disturbances, osteoporosis, bone fractures, and mortality [7], meta-analysis suggests that SNa levels higher than 132–135 mmol/L will decrease morbidity and mortality [8,9].

The aim of this study was to propose an easy way to determine solute and fluid intakes in patients with SIADH, at least in those whose urine osmolality is higher than their serum osmolality. To do so, we measured urine creatinine concentration, which allowed for the calculation of fractional urine solute excretion (FE.Osm in %) and fractional urine volume excretion (V/eCcr in %). Both give an easy estimation of daily solute and fluid intakes.

Patients with low FE.Osm (<1.4%) and low diuresis (V/eCcr % < 0.8%), usually meaning an estimated solute intake lower than 500 mmol/day and a diuresis lower than 1 L/24 h, will not have a significant response to mild water restriction and will be better treated by an increase in solute intake.

Similarly, patients with high FE.Osm (>2.5% or >900 mmol/day), if associated with high diuresis (V/eCcr > 1.5%, usually representing a urine volume >2 L/24 h), could be treated by mild WR (<1.5–2 L/24 h). A simple measurement of the creatinine concentration on a morning urine spot sample can help to estimate the solute and fluid intakes of patients with chronic hyponatremia secondary to SIADH. However, the best way to estimate the fluid and solute intakes is by a complete 24 h urine collection.

## 2. Materials and Methods

Table 1 explains the sample in which the estimated solute output and urine volume were calculated, including controls (*n* = 35) and patients with chronic hyponatremia due to SIADH (*n* = 65). We first compared the data to those obtained in the control patients followed in the out patients clinics who were without diabetes and who had normal renal function. Only control patients with a urine osmolality higher than their serum osmolality were included to verify if the calculated estimation of solute intake (obtained by a morning urine spot sample) was similar to the one obtained by measurements from a 24 h urine collection.

We retrospectively collected the data of the last 65 consecutive patients with classical criteria of SIADH in whom urine osmolality and urine sodium and creatinine concentrations were available on a morning urine spot sample.

All these patients suffered from hyponatremia for at least one week or even several weeks, as was the case for most of them. For patients to be included, their serum sodium concentrations had to be stable, with a minimal daily variation of 1–3 mmol/L before any treatment.

We also only included asymptomatic patients with a SNa concentration in the range of 120–132 mmol/L. Patients with diabetes and/or abnormal renal function were excluded.

All these patients were on their usual fluids and diet before any treatment (WR with or without urea).

We only included patients with a urine osmolality higher than their serum osmolality and with urine sodium concentrations higher than 30 mmol/L. In 30 patients, a complete 24 h urine collection was available, on another day, to allow us to see whether the calculated solute intake and calculated urine volume corresponded with the real measurements that were obtained by the 24 h urine collection (see Table 2). When precise urine collections were not available, the MDRD formula was used to estimate glomerular filtration.

Statistical analysis was performed with GraphPad Software (GraphPad Inc, La Jolla, CA, USA). The Kolmogorov–Smirnov test was applied to determine if the analyzed variables were normally distributed. The comparison between the groups was performed with a Student’s *t* test or a paired *t* test. Linear regression was also used. Data are presented as mean ± SD.

This retrospective study was approved by the Ethics Committee of our hospitals.

## 3. Results

**Figure 1:**
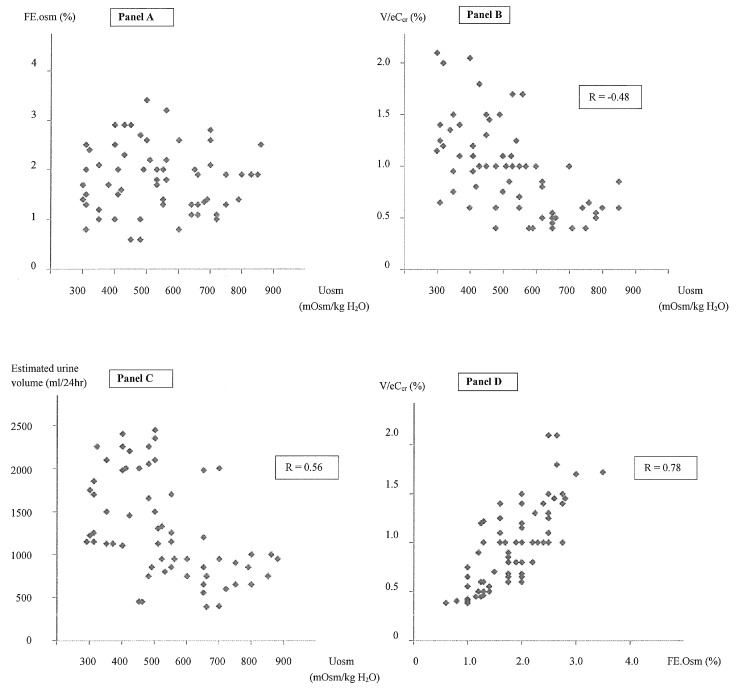
Panel 1A-Lack of correlation between Uosmolality and FE.Osm. Panel 1B-Mild correlation between urine osmolality and V/cCcr (%) (*r* = 0.48; *p* < 0.001). Panel 1C-Correlation between urine osmolality and estimated urine volume (*r* = 0.56, *p* < 0.001). Panel 1D-High correlation between FE.Osm and V/eCcr (%) (*r* = 0.78; *p* < 0.001).

**Figure 2:**
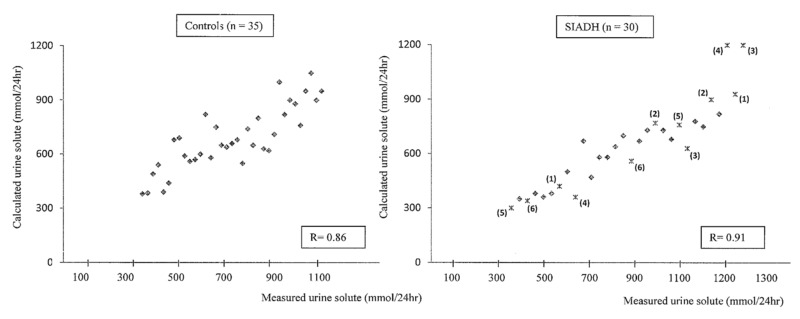
Correlation between the calculated urine solute output and the measured values in 35 controls and 30 patients with chronic hyponatremia due to SIADH. In six patients, represented by a number in brackets, the values are given before and after treatment with urea (see Table 3). Calculated urine solute (mmoL/24 h) was obtained by the formula using FE.Osm (see Table 1).

**Figure 3:**
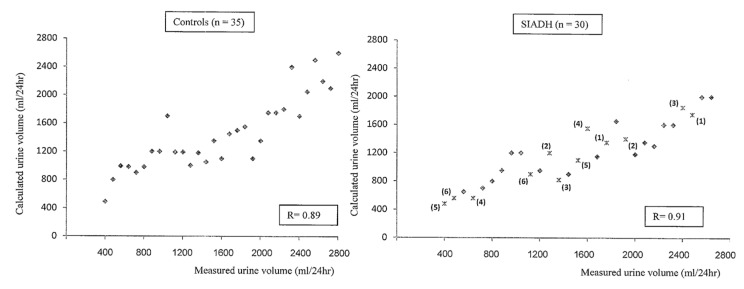
Correlation between the calculated urine volume and the measured values in 35 controls and 30 patients with chronic hyponatremia due to SIADH. In six patients, represented by a number in brackets, the values are given before and after treatment with urea (see Table 3).

In the 35 non-hyponatremic controls, whose age (55 ± 18 years) and sex distributions were (15 M; 30 F) similar to those of our patients with SIADH (age 61 ± 17 years; sex distribution 28 M; 37 F), we calculated a urine osmoles output of 700 ± 180 mmol/24 h, corresponding fairly well with the 24 h directly measured urine osmoles output (720 ± 220 mmol/24 h). As expected, we observed that FE.Osm, indirectly reflecting the solute intake, is not correlated with urine osmolality and that urine osmolality is weakly correlated with V/eCcr (%) (*r* = −0.48) or the estimated urine volume (*r* = −0.56) (Figure 1). For example, with the same urine osmolality of 475 mOsm/kg H2O, a broad variation of V/eCcr values between 0.4% and 1.5% were observed in four patients, meaning an estimated urine volume between 470 mL and 2200 mL. The measured urine solute output or urine volume was highly correlated with the calculated value in the control and SIADH patients (*r* = 0.86 to *r* = 0.91) (Figure 2 and Figure 3). The calculated total solute output in the SIADH patients (*n* = 65) was similar to the one measured in the controls (650 ± 270 mmol/24 h) (NS). The measured solute output in the 24 patients with SIADH, where it was available, was similar to that in the controls (588 ± 188 mmol/24 h; NS) (excluding the six patients under urea therapy, see later).

**Figure 4:**
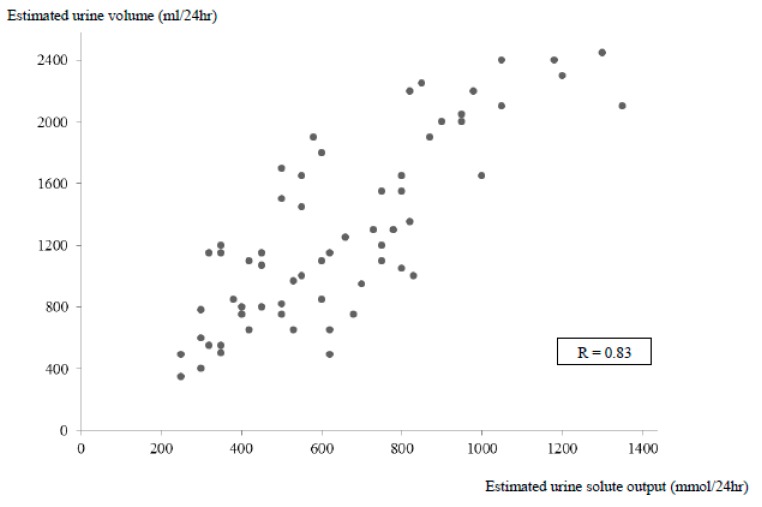
Correlation between the estimated solute output and the estimated urine volume in 65 patients with SIADH.

Further, 13 patients presented an estimated daily urine volume ≥2000 mL, associated with a high solute intake (>900 mmol/24 h in 10 patients; Figure 4) and 19 patients presented a daily low solute intake <500 mmol, associated with a low urine volume (<1000 mL/24 h in 13 patients).

In our control group with exact 24 h urine collection, the difference between the measured and calculated solute or urine volumes never exceeded 20% (data not shown). In our 30 patients with SIADH, the difference between the measured or calculated values of urine volume or solute output never exceeded 22% (data not shown).

In normal subjects with urine osmolality higher than serum osmolality, we also observed a correlation between FE.Osm and V/eCcr (%), however this was less significant (y = 0.017 + 0.605 x; *r* = 0.58; *p* < 0.001) (data not shown) than in the SIADH patients (y = 0.025 + 0.519 x; *r* = 0.78; *p* < 0.001) (Figure 1). As expected, FE.Na was correlated with FE.Osm (y = 0.156 + 0.455 x; *r* = 0.66) or with diuresis expressed by V/eCcr (*r* = 0.64), however diuresis was better correlated with FE.Osm (*r* = 0.78) (Figure 1).

Table 2 shows the data of three patients. The first patient (J.P.) had idiopathic SIADH with chronic hyponatremia for many months previously. For this patient, the calculated solute intake and calculated urine volume were similar to the real measurements. The FE.Osm was 1.7% and the V/eCcr was low at 0.62%, meaning an estimated urine volume of 795 mL (0.089 L/min × 1440 × 0.0062 = 0.795 L/24 h). If we assume that fluid intake was between 800 mL and 1100 mL (urine volume + 300 mL), it is easy to see that it will be difficult to comply with more strict water restriction in the long term.

When we added 30 g of urea orally to the mild water restriction (<1.5 L/day), the SNa values normalized and the daily urine volume increased to a calculated amount of 1530 mL (0.089 × 1440 × 0.012 = 1.53 L/24 h), while we measured it at 1350 mL (data presented after one month of treatment). With a supplemental oral load of 500 mmol adding 30 g of urea to the usual solute intake, we can estimate the total solute output at about 1230 mmol (291 × 0.089 × 1440 × 0.033), and we measured it at 1025 mmol/24 h (if we use the formula: eCcr (L/min) × 1440 × V/eCcr × urine osmolality, the value is 1161 mmol/24 h). Fluid intake was estimated to be 1350−1650 mL/day, which is much more comfortable. The second patient also had idiopathic SIADH with a high FE.Osm (2.8%) and a high V/eCcr of 1.9%. Estimated solute intake was 820 mmol, and estimated urine volume was 2025 mL; we measured, respectively, 1005 mmol for solute intake and 2500 mL for urine volume, implying a fluid intake of around 2500–2800 mL/day. In this patient, the measured creatinine clearance was 91 mL/min, while the MDRD value gave a much lower value of 74 mL/min. This contributes to the difference between the measured and the calculated values of urine solute excretion and fluid intake. Fluid intake was estimated to be around 2300 mL/day (see Table 2), and mild water restriction (<2L/day) normalized SNa in this patient.

The third patient (V.D.) also presented a urine osmolality similar to that of the second patient (H.M.) (420 mOsm/kg H2O) and a urine sodium concentration of 60 mmol/L (also similar to that of the second patient) (see Table 2). SIADH in this patient was due to the antiepileptic drug carbamazepine, and FE.Osm was low at 0.96%, corresponding to an estimated solute intake of 365 mmol/day if calculated with the formula eCr (L/min) × Sosm × 1440 × FE.Osm (in %) and a value of 363 mmol/day if calculated with eGFR (L/min) × 1440 × V/eGFR (in %) × urine osmolality. We measured 336 mmol/day. An estimated urine volume of 866 mL was calculated, and we measured 800 mL. After two weeks of mild water restriction, the SNa level increased by 2 mmol/L, solute intake was estimated to be similar at 363 mmol/day, and urine volume was estimated to be 582 mL/day (values similar to the measured values). The intake of 15 g urea normalized SNa (137 mmol/day). The solute intake was estimated to be 686 mmol/day or 790 mmol/day (we measured 788 mmol/day), and the daily urine volume was estimated to be 922 mL (we measured 920 mL/day), implicating a fluid intake of around 900–1200 mL/day. The urine osmolality increased to 857 mOsm/kg H2O during urea therapy in this patient.

**Figure 5:**
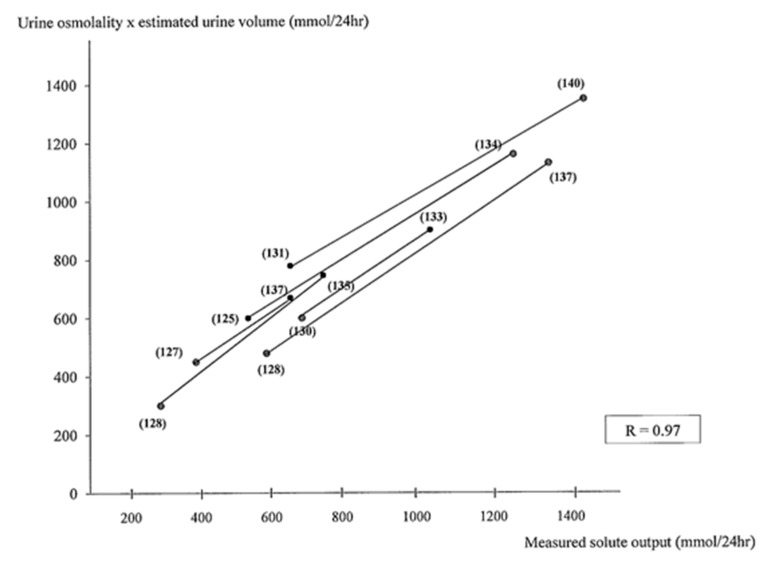
Evolution of SNa in six patients with chronic hyponatremia related to SIADH treated by urea (2 patients with 15 g/day and 4 patients with 30 g/day) (see Table 3). Estimated urine volume was obtained by the formula: *eCcr* × *1440* × *S.Creat / U.Creat.100 in %* (y = –38.3 + 1.035 x; *r* = 0.97; *p* < 0.001).

In six patients treated with oral urea, we had the opportunity to compare the evolution of the values obtained by exact measurements with the evolution of the calculated values (see Table 3 and Figure 2 and Figure 3, when calculated with formula FE.Osm, and Figure 5).

Whether estimated solute excretion was calculated by the formula *eGFR (L/min) w Sosm* × *1440* × *FE.Osm* or by the formula *eGFR (L/min)* × *1440* × *V/eCcr (%) estimated 24 h urine volume* × *urine osmolality*, both results were strongly correlated (y = 16.6 + 0.97 x; *r* = 0.94).

In the 24 patients with SIADH where urine collection was available (excluding the six patients treated with urea), we also found a strong correlation between the measured solute output and the estimated solute output calculated by the formula *estimated urine volume* × *by urine osmolality* (y = 45 + 0.89 x; *r* = 0.93). Figure 5 shows the evolution of the six patients with chronic SIADH before and during urea therapy. The estimated urine volume value was calculated by the formula *eCcr (L/min)* × *1440* × *S.Creat/U.Creat (in %)*. The measured or calculated solute output was, as expected, strongly correlated in these patients.

## 4. Discussion

Solute balance (mainly of electrolytes) and water balance are dissociated in normal subjects. Once urine osmolality is higher than serum osmolality, a high solute intake will be associated with an increase in diuresis; the same relationship, but stronger, is observed in SIADH. In the steady state when both fluid and solute intakes are stable, patients with SIADH have a relatively stable SNa level and stable urine osmolality, although it differs from patient to patient, probably due to different ADH levels, different individual escape to ADH, different concentration abilities of the kidney, etc. [7].

It is interesting to note that whatever treatment is applied, once SNa is normalized, the endogenous secretion of ADH increases to avoid dehydration [6]. This explains the increase in urine osmolality observed during treatment by WR or WR combined with urea [2,6]. In patients with SIADH whose urine osmolality is higher than their serum osmolality (which is usually the case), our data suggest that the measurement of urine creatinine in addition to urine osmolality allows an easy estimation of solute and fluid intakes. A low FE.Osm (<1.4%) usually reflects a low solute intake (<500 mmol/24 h). Low solute intake, if associated with high fluid intake (>2 L/24 h), could theoretically be treated by mild WR (<1.5 L/day). We know that low solute intake (LSI) contributes to hyponatremia in patients with mild dilution deficit and relative polydipsia [10]. These patients will be particularly easy to treat with small doses of urea (15 g/day, representing 250 mmol) if they do not accept some restriction in fluid intake. Patients with high solute intake (FE.Osm >2.5% or an estimated solute intake >900 mmol/day) and with high fluid intake (estimated >2 L/day) will usually respond to mild water restriction (see H.M. in Table 2).

An alternative treatment for hyponatremia due to SIADH is to progressively increase the dosage of urea until SNa is normalized, without the association of mild water restriction. In a recent series of patients with SIADH associated with cancer, 90% normalized their SNa level, despite free water intake, but needed a daily dose of 30–60 g urea [11]. Even though the cost of urea is low, we think that mild water restriction is helpful (<1.5–2 L/day) [12]. In different studies from the literature on vaptans in hyponatremia secondary to SIADH, mild water restriction was associated with V2 antagonists. If free water intake was allowed, only 50% of patients normalized their SNa [1,12].

The knowledge of the usual levels of solute and fluid intakes in a patient will allow a more rational approach to their treatment: WR alone or combined with some increase in solute intake (usually 15 g or 30 g urea daily) [13] (see Table 4). Increasing the daily solute intake in the form of NaCl can also be performed if the dose needed is not too high and if urine osmolality is also not too high (<500 mOsm/kg). In our experience, if we increase salt intake by 9 g NaCl (310 mmol/L), patients usually do not tolerate it very well (gastric symptoms and hydrosaline retention) [14]. It is known that an acute salt load cannot be well excreted if urine is highly concentrated [15]. Urea therapy is associated with a mild body weight decrease during initiation, while with a high salt supplement, body weight increases [14]. If we want to increase solute intake by means of diet, we need to increase salt and protein intakes to high levels. We recall that 10 g of protein will produce 50 mmol of urea. According to this principle, patients would have to eat 50 g of protein more than their usual daily quantity to produce an extra 250 mmol of urea. In terms of meat, this would correspond to 165 g to 250 g of extra meat, assuming that meat has a 20–30% protein content, depending on the type of meat. In combination with an extra 4.5 g load of NaCl (about 155 mmol), this would represent a substantial extra solute load of 400 mmol daily and would increase diuresis by 1 L if urine osmolality is fixed to, e.g., 400 mOsm/kg H2O.

In the long term, especially in elderly persons, an increase in solute intake by means of urea (15 g or 30 g daily) is much safer to ensure sufficient daily solute intake.

The difference between the measured and calculated values is due to different factors. The main factor is likely the difference in daily solute intake (mild variations in salt and meat intakes; these parameters were not controlled, but variations are likely small, maybe around 10–20%). It could also only be applied to patients with stable chronic hyponatremia. In SIADH, we know that during the development of hyponatremia, salt excretion exceeds intake for a few days [7]. The suggested methods will overestimate solute intake during this period. The difference between eGFR and real GFR could also be a contributing factor.

If a 24 h urine collection is available in a patient with stable chronic hyponatremia, we can also easily establish their solute and fluid intakes and adapt our therapeutic attitude: mild WR with or without increasing their solute intake [16]. These patients could of course also be treated with a vaptan [17,18,19].

## Figures and Tables

**Table 1:** Formula used to estimated daily solute intake, urine volume and water intake.

- eGFR estimated by serum creatinine and MDRD
- FE.Osm = C osm/Ccr (%) = $\frac{\mathrm{Uosm}}{\mathrm{Sosm}}$ × $\frac{S.\mathrm{Creat}}{U.\mathrm{Creat}}$ × 100 in % (*N* = 2.0 ± 0.5%) *
- FE Na = $\frac{\mathrm{Urine}\;\mathrm{Na}}{\mathrm{Serum}\;\mathrm{Na}}$ × $\frac{S.\mathrm{Creat}}{U.\mathrm{Creat}}$ × 100 in % (*N* = 0.8 ± 0.35%) *
- Estimation of solute intake: GFR (L/min) × S osm × 1440 × FE.Osm (%) (mmol/24 h)
- Estimation of solute intake: urine osmolality × estimated 24 h urine volume
- Real solute intake: Urine volume (L/24 h) × Urine osmolality (*N* = 700 ± 200 mmol/24 h) *
- Estimation of 24 h urine volume:
- V/Ccr (%) = Pcr/U.Creat × 100 (%)
- eGFR (L/min) × 1440 × V/Ccr (%) = L/24 h
- Estimation of fluid intake: Urine volume (+300 mL)

* Normal value in our hospital.

**Table 2:** Estimation of fluid and solute intakes in three patients with hyponatremia related to SIADH and treated by water restriction (WR) with or without urea (solute excretion) where calculated by two different methods ^(1),(2)^.

	J.P.		H.M.		V.D.		
	T0	WR + 30 g/day Urea-Day 30	T0	WR (<2 L/day) Day 30	T0	WR (<1.5 L/day) Day 15	WR + 15 g/day (<1.5 L/day) Day 30
SNa/Una (mEq/L)	125/142	133/72	129/55	135/61	127/60	129/132	137/176
Sosm/Uosm (mOsm/kgH2O)	265/754	291/759	262/402	292/398	268/420	263/778	282/857
S.Creat/U.Creat (mg/dL)	0.9/145	0.9/74	0.8/42	0.8/52	0.8/135	0.8/183	0.9/124
Measured creatinine Clearance (mL/min)	84	77	91	90	94	89	91
MDRD	89	89	74	74	102	102	89
FE.OsmOsm (%)	1.7	3.3	2.8	2.5	0.92	0.96	1.9
V/Ccr (%)	0.62	1.2	1.9	1.5	0.59	0.43	0.72
Estimated solute excretion ^(1)^ (mmol/24 h)	577	1230	820	778	365	363	686
Estimated solute excretion ^(2)^ (mmol/24 h)	599	1161	814	636	363	452	790
Estimated urine volume (mL/day)	795	1530	2025	1600	866	631	922
Measured urine solute output (mmol/day)	565	1025	1005	796	336	435	788
Measured urine volume (mL/24 h)	750	1350	2500	2000	800	560	920

^(1)^ Obtained by eGFR (L/min) × Sosm × 1440 × FE.OsmOsm (in%) (mmol/24 h). ^(2)^ Obtained by eGFR (L/min) × 1440 × V/eCcr (%) × urine osmolality (mmol/24 h).

**Table 3:** Some biochemical parameters in six patients with chronic hyponatremia secondary to the inappropriate secretion of antidiuretic hormone (SIADH) before and after at least one month of treatment with urea (15 to 30 g/day) and comparing exact urine values with calculated values.

	T_0_	T _(≥30 days)_ Urea Treatment
SNa (mEq/L)	128 ± 2	137 ± 2.5 *
FE.Osm (%)	1.58 ± 0.57	3.0 ± 0.56 *
V/Creat Clear (%)	0.66 ± 0.23	1.2 ± 0.27 *
Real urine volume (mL/24 h)	912 ± 315	1636 ± 310 *
Estimated urine volume (mL/24 h)	875 ± 250	1524 ± 300 *
Measured urine solute (mmol/24 h)	578 ± 170	1038 ± 215 *
Calculated urine solute ^(1)^ (mmol/24 h)	537 ± 183	1027 ± 234 *
Calculated urine solute ^(2)^ (mmol/24 h)	470 ± 144	1063 ± 240 *
Urine osmolality (mOsm/kgH_2_O)	594 ± 155	709 ± 99 (NS)

* *p* < 0.01; FE.Osm: 2 ± 0.5% (Normal value); Normal 24 h urine solute output: 720 ± 220 mmol/24 h; ^(1)^ Obtained by eGFR (L/min) × Sosm × 1440 × FE.Osm (in%) (mmol/24 h); ^(2)^ Obtained by eGFR (L/min) × 1440 × V/eCcr (%) × urine osmolality (mmol/24 h).

**Table 4:** Suggested therapeutic implication of the measurement of FE.OsmOsm (%) and V/eCcr (%) in chronic hyponatremia related to SIADH.

FE.OsmOsm <1.4% ^(1)^ V/eCcr <0.8% ^(1)^	FE.OsmOsm >1.4–2.5% ^(2)^ V/eCcr >0.8–1.5% ^(2)^	FE.OsmOsm >2.5% ^(3)^ V/eCcr >1.5% ^(4)^
Treatment:	Treatment:	Treatment:
Increase daily solute intake (for ex. Urea)	Increase daily solute intake (15–30 g urea) combined with mild water restriction (<1.5–2L/day)	Water restriction (<1.5–2 L/day)

^(1)^ Or measured solute output <500 mmol/24 h and diuresis <1 L/24 h; ^(2)^ Or measured solute output between 500–900 mmol/24 h and diuresis between 1–2 L/24 h; ^(3)^ Or measured solute output >900 mmol/24 h and diuresis >2 L/24 h; ^(4)^ Patients with high diuresis (>2 L/24 h) should always first be treated by simple water restriction.
